# Expression patterns of *circRFX3* and *miR-587* in colorectal cancer patients

**DOI:** 10.22099/mbrc.2025.52016.2080

**Published:** 2025

**Authors:** Samaneh Najafi, Zivar Salehi, Farhad Mashayekhi, Hamid Saidi-Saedi

**Affiliations:** 1 Department of Biology, University Campus 2, University of Guilan, Rasht, Iran; 2 Department of Biology, Faculty of Sciences, University of Guilan, Rasht, Iran; 3 Department of Internal Medicine, School of Medicine, Guilan University of Medical Sciences, Rasht, Iran

**Keywords:** circRFX3, miR-587, CRC, Gene expression, Real-time PCR

## Abstract

Circular RNAs (circRNAs) are non-coding, single-stranded RNAs considered by their closed-loop structures. Research has established a connection between circRNAs and cancer progression. The objective of this project was to evaluate the expression levels of a newly discovered circRNA, *circRFX3 *(*hsa_circRFX3_003*), along with its target gene, *miR-587*. The study involved 60 patients diagnosed with Colorectal cancer (CRC) and 60 healthy individuals as controls. Total RNA was extracted from blood samples, converted into cDNA, and analyzed using qRT-PCR. The findings revealed an up-regulation of *miR-587* and a down-regulation of *circRFX3* in the blood samples of CRC patients. An inverse relationship was observed between the levels of *miR-587* and *circRFX3*; however, there was no significant difference in circRFX3 expression levels between stages I+II and stages III+IV. The levels of miR-587 expression were linked to tumor size and location. Both *circRFX3* and *miR-587* play significant roles in the pathophysiology of CRC; however, additional research is necessary to elucidate their specific contributions to CRC development.

## INTRODUCTION

Colorectal cancer (CRC) is the third most prevalent type of cancer and the second primary cause of death due to cancer. In Iran, it is the fourth most common cancer affecting both men and women [1]. 

Circular RNAs (circRNAs) are defined by their covalently closed configuration, lacking both 5′ end caps and 3′ poly(A) tails. Research has uncovered a diverse array of functions for circRNAs, including their ability to translate into polypeptides and act as sponges for microRNAs (miRNAs). CircRNA Regulatory Factor X3 (*circRFX3*), also known as *hsa_circ_ 0001836*, comprises 111 nucleotides and is situated at the chromosomal location 9p2.24. Research has demonstrated a significant upregulation of *circRFX3* in glioblastoma cell lines [2]. Additionally, another study reported elevated levels of *circRFX3* in glioma tissues when compared to adjacent normal tissues [3]. Nevertheless, there is insufficient research investigating the potential correlation between *circRFX3* and *miR-587* gene expression levels in the context of CRC development.

MicroRNAs are small, non-coding RNA molecules that range from 18 to 25 nucleotides in length and play a crucial role in the regulation of gene expression at the post-transcriptional stage. The progression of cancer is closely linked to the dysregulation of miRNA expression. A rise in *miR-133a *expression has been observed in breast cancer [4]. Additionally, a rise in *mir-142 *expression has been observed in Papillary thyroid carcinoma [5]. *MicroRNA-587*, referred to as *miR-587*, is crucial in the advancement of multiple cancers. 

The pathophysiology of colorectal cancer remains inadequately elucidated, and recent investigations have focused on the significance of regulatory RNAs. Consequently, the aim of this project was to assess the levels of *circRFX3* and *miR-587* expression in blood samples of CRC patients. Additionally, it explored the correlations between the clinicopathological features of CRC and the levels of these specific genes expression.

## MATERIALS AND METHODS

### Sampling:

This case-control investigation involved a comparison between 60 patients diagnosed with CRC (28 females and 32 males) and 60 healthy individuals, which included 30 females and 30 males. Blood samples were obtained from Razi Hospital in Rasht, Iran, during the period from January 2023 to November 2023. The diagnosis of CRC was made by a qualified gastroenterologist and subsequently validated through pathological analysis of tissue samples. The criteria for inclusion in the study were: 1) Age between 50 and 80 years; 2) Confirmation of CRC through pathological assessment; and 3) Selection of healthy subjects devoid of any history of diabetes, cancer, viral infections, or chronic illnesses, including cardiovascular and liver diseases. The exclusion criteria encompassed: 1) Individuals with other medical conditions such as diabetes, Crohn's disease, ulcerative colitis, Lynch syndrome, or various polyposis syndromes; 2) Individuals who had undergone prior chemotherapy or radiotherapy; and 3) Individuals with a history of colectomy.

### Extraction of RNA and cDNA synthesis:

Lymphocytes were separated from peripheral blood using Ficoll-Lymphodex (Inno-train, Germany). Total RNA extraction from the lymphocytes was performed with RNX PLUS (Sinaclon, Iran), following the manufacturer's protocol. The extracted RNA was stored in RNase-free tubes at -70°C in preparation for cDNA synthesis, and its concentration was measured using the NanoDrop EzDrop 1000 (Blue-Ray Biotech, Taiwan). A reverse transcription reaction for cDNA synthesis was performed following the instructions provided by the manufacturer, using a cDNA Synthesis Kit from Sinaclon, Iran. The reaction conditions required incubation at 50°C for 50 minutes, followed by a final step at 85°C for 5 minutes. The specific primer for *miR-587* was 5’-GTCGTATCCAGTGCAGGGTC CGAGGTATTCGCACTGGATACGACGTGACT-3’. Oligo 7 software (Oligo v7.60, CO, USA) was used for primer design.

### Real-Time PCR:

The primers employed in the qRT-PCR analysis for *circRFX3*, *miR-587*, and *U6* are detailed in Supplementary Table 1 and were produced by Metabion Co. (Bavaria, Germany). The Quantitative PCR was conducted by specific primers, SYBR green (Sinaclon, Iran), and the Roche LightCycler® 96 device (Roche, Germany) to quantify gene expression. *U6* served as the reference gene. The process involved denaturing for 20 seconds at 95°C, annealing for 30 seconds at 58°C, and extending for 30 seconds at 72°C, carried out in triplicate with a total volume of 25 µl over 40 cycles. The data were evaluated using the 2^-∆∆Ct^ method.

### Statistical analysis:

 The outcomes were expressed as the mean ± standard deviation. A one-way ANOVA was applied for the purpose of making multiple comparisons, while an independent two-sample t-test was used to contrast the two groups. Pearson’s correlation coefficient was employed to evaluate the association between *circRFX3* and *miR-578*. To explore the potential clinical diagnostic relevance of the selected circRNA and miRNA, receiver operating characteristic (ROC) curve analysis was conducted. Furthermore, logistic regression was utilized to determine risk factors. A *p*-value of under 0.05 was regarded as statistically significant. All statistical evaluations were performed using GraphPad Prism 8.

## RESULTS

Table 1 presents the demographic details of the patients involved in the study. The mean age of the patients was approximately 68±8.9 years, while the healthy volunteers had a mean age of 64 ± 10.5 years. Statistical analysis revealed no significant differences in age or gender between the patient cohort and controls (*p*>0.05). 

**Table 1:** Logistic regression analysis and correlation between clinicopathological data and *miR-587 *and *circRFX3* expression in CRC

**Characteristics**	*** n*** ** (%)**	**OR**	**95% CI**	***p*** **-value**
**Gender**				
Male	32 (53.33)			
Female	28 (46.66)	1.14	0.56-2.34	0.71
**Age of diagnosis (years)**				
<60	27 (45.0)			
≥60	33 (55.0)	0.51	0.25-1.05	0.07
**Smoking status**				
Never	40 (66.66)			
Past or current use	20 (33.33)	0.53	0.26-1.12	0.10
**Change in bowel habi** **ts**				
Positive	39 (65.0)			
Negative	21 (35.0)	4.70	2.17-10.17	<0.01
**Rectal bleeding**				
Positive	19 (31.66)			
Negative	41 (68.33)	13.44	3.00-60.89	<0.01
**Anemia**				
Positive	51 (85.0)			
Negative	9 (15.0)	5.30	2.22-12.67	<0.01
**Tumor location**				
Colon	41 (68.33)			
Rectum	19 (31.66)	2.13	0.04-111.2	0.71
**Tumor size**				
≤ 4 cm	23 (38.33)			
> 4 cm	37 (61.66)	0.63	0.01-32.67	0.82
**Stage**				
I+II	45 (75.0)			
III+IV	15 (25.0)	0.34	0.01-17.91	0.59

**Figure 1:**
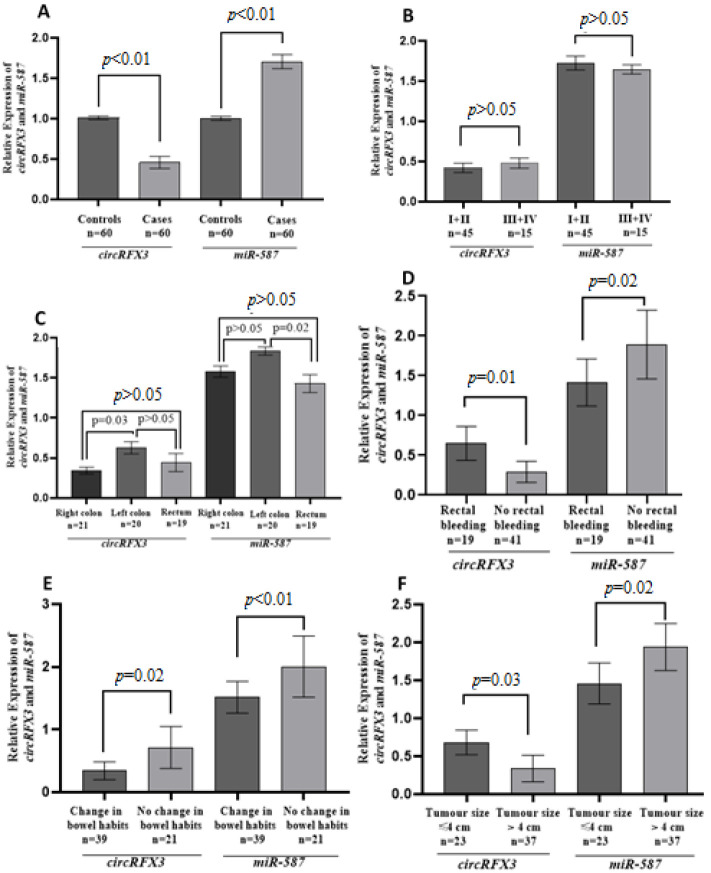
Expression pattern of *circRFX3* and *miR-587*. (A) Relative expression of the genes in CRC cases and controls, (B-F) relative expression of *circRFX3* and *miR-587 *in different clinicopathological features.

Notably, the expression of *CircRFX3 *was shown to be meaningfully lesser in patients compared to controls, with a mean relative expression of 0.46 ± 0.07 (Fig. 1A). Furthermore, the research indicated that *circRFX3* expression was associated with various clinical parameters, including disease stage (Fig. 1B), tumor location (Fig. 1C), presence of rectal bleeding (Fig. 1D), changes in bowel habits (Fig. 1E), and tumor size (Fig. 1F). 

**Figure 2:**
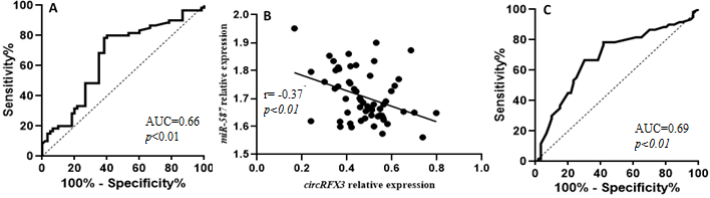
ROC curves and Correlation analysis. (A) Receiver operating characteristic analysis of *circRFX3 *in peripheral blood lymphocytes from CRC. (B) Correlation of *circRFX3 *expression with *miR-587* in CRC. *p*<0.05 was considered statistically significant. (C) Receiver operating characteristic analysis of *miR-587* in peripheral blood lymphocytes from CRC.

There was no significant difference in circRFX3 expression levels between stages I+II and stages III+IV (*p*>0.05). It was more downregulated in right-sided cancer than in both rectal and left-sided cancer. *CircRFX3* was more downregulated in CRC with no rectal bleeding than in CRC with rectal bleeding. This circRNA was more downregulated in CRC patients with changes in bowel habits than in patients without changes in bowel habits. It was more downregulated in CRCs with tumors larger than 4 cm than in CRCs with tumors equal to or less than 4 cm in size. The study also evaluated the diagnostic potential of *CircRFX3* in differentia-ting CRC patients from controls, yielding an area under the curve (AUC) of 0.66 with a 95% confidence interval (CI) of 0.56-0.76. These results show that *CircRFX3* may be used as a predictive biomarker for distinguishing CRC from the control group (Fig. 2A).

*CircRFX3* was shown to play as a sponge for *miR-587* [2]. Furthermore, data from circinteractome analysis indicate that *circRFX3* and *miR-587* possess complementary sequences. Based on these observations, *miR-587* was chosen as the target of *circRFX3* in this research. In CRC patients, *miR-587* expression was shown to be meaningfully elevated compared to the controls, with a mean relative expression of 1.70 ± 0.09 (Fig. 1A). Additionally, *miR-587* expression was related to various clinical parameters, including cancer stage (Fig. 1B), the location (Fig. 1C), presence of rectal bleeding (Fig. 1D), changes in bowel habits (Fig. 1E), and the size (Fig. 1F) of tumor, as revealed by the study. *MiR-587* was more upregulated in CRC with stages I+II than in CRC with stages III+IV. It was more upregulated in left-sided cancer than in both rectal and right-sided cancer. *MiR-587 *was more upregulated in CRC with no rectal bleeding than in CRC with rectal bleeding. This miRNA was more upregulated in CRC without changes in bowel habits than in CRC with changes in bowel habits. It was more upregulated in CRCs with tumors larger than 4 cm than in CRCs with tumors equal to or less than 4 cm in size. 

A negative relationship was noted between the levels of *miR-587* and *circRFX3* in patients with CRC (Fig. 2B). The research investigated the diagnostic efficacy of *miR-587* in differentiating the CRC patients from the controls, yielding an AUC of 0.69 with a 95% CI of 0.59 to 0.78 (Fig. 2C). This suggests that *miR-587* might be used as a valuable biomarker for distinguishing CRC from the control population. The results of the logistic regression analysis, including odds ratios and 95% confidence intervals, are presented in Table 1. Notable differences were identified between the case and control groups regarding alterations in bowel habits, rectal bleeding, and anemia, with statistical significance (*p*<0.05). Conversely, there was no significant changes in other characteristics between the groups.

## DISCUSSION

A number of circRNAs have been recognized as having central regulatory roles in the onset and development of tumors. Prior research has indicated that the upregulation of *circ-SMAD7* serves to inhibit both cell immigration and invasion in CRC [6]. Furthermore, overexpression of *circ_0137008* was demonstrated to inhibit the CRC cell proliferation [7]. 

In the present study, we selected *circRFX3* as an experimental subject that has never been investigated before in CRC. The results of this study showed that *circRFX3* expression was meaningfully decreased in CRC cases. Subsequent analysis confirmed a notable relationship between the expression level of *circRFX3* and both tumor staging and its anatomical location.

Studies have demonstrated that *circRFX3* acts as a sponge for *miR-1179* and *miR-1229* in glioma [3]. *MiR-1229* plays significant roles in the advancement of various tumors, including colorectal cancer and gastric cancer [8, 9]. Furthermore, it was suggested that *circRFX3* enhances PDIA3/β-catenin pathway in glioblastoma while also sponging *miR-587* [2]. Consequently, as part of this investigation, we assessed the expression levels of *miR-587*, which is targeted by *circRFX3*. Our findings indicated that *miR-587* is overexpressed in the blood samples of CRC patients. Moreover, a significant correlation was identified among the expression of *miR-587* and specific clinicopathological characteristics of the patients. We also observed a negative correlation between the levels of expression of *miR-587* and *circRFX3*.

Increasing evidence suggests that abnormal levels of *miR-587* might be associated with cancer development. For example, elevated *miR-587* expression was observed in cervical cancer [10]. Research indicates that *miR-587* promotes the expression of the *CYLD* gene, thereby contributing to the lung carcinoma development [11]. 

First of all, the number of participants in our study was quite limited, indicating the need for further research with a larger cohort. Another limitation is the absence of tissue sample analysis; future studies should incorporate this aspect for a more comprehensive understanding. Furthermore, both environmental and genetic factors were shown to be involved in the development of CRC. Our research focused solely on genetic factors, highlighting the importance of conducting gene-environment interaction analyses to gain deeper insights into the patho-mechanisms associated with CRC.
