# Supplementary material for: Expression patterns of circRFX3 and miR-587 in colorectal cancer patients
Source: Mol Biol Res Commun. 2025;14(3):243–8. doi: 10.22099/mbrc.2025.52016.2080 (PMC12046363; doi:10.22099/mbrc.2025.52016.2080)
Supplement: Supplementary file 1 — Tables S1 [file MBRC-14-243-s001.pdf]

Table S1: Sequences of primers used in this study.

Name	Sequence (5'-3')	Produce size (bp)
circRFX3 forward	TATGTA AATGATGGGGGTGGAGA	70
circRFX3 reverse	TTCCATAGCATTGACAACCATCT	
miR-587 forward	GCGCCGTTTCCATAGGTGATGA	66
miR-587 reverse	ATCCAGTGCAGGGTCCGAG	
U6 forward	CTCGCTTCGGCAGCACA	94
U6 reverse	AACGCTTCACGA ATTTGCGT	
